# PAI-1 Exacerbates White Adipose Tissue Dysfunction and Metabolic Dysregulation in High Fat Diet-Induced Obesity

**DOI:** 10.3389/fphar.2018.01087

**Published:** 2018-09-26

**Authors:** Lin Wang, Liyuan Chen, Zheran Liu, Yaofang Liu, Mao Luo, Ni Chen, Xin Deng, Yulin Luo, Jing He, Liping Zhang, Michael A. Hill, Rong Li, Jianbo Wu

**Affiliations:** ^1^Drug Discovery Research Center, The School of Pharmacy, Southwest Medical University, Luzhou, China; ^2^Laboratory for Cardiovascular Pharmacology of Department of Pharmacology, The School of Pharmacy, Southwest Medical University, Luzhou, China; ^3^Queen Mary School, Medical College of Nanchang University, Nanchang, China; ^4^Department of Gynecology and Obstetrics, The Affiliated Hospital of Southwest Medical University, Luzhou, China; ^5^Dalton Cardiovascular Research Center, University of Missouri, Columbia, MO, United States; ^6^Department of Medical Pharmacology and Physiology, University of Missouri, Columbia, MO, United States

**Keywords:** PAI-1, inflammation, macrophage, adipose tissue, high-fat diet

## Abstract

**Background:** Plasminogen activator inhibitor (PAI)-1 levels and activity are known to increase during metabolic syndrome and obesity. In addition, previous studies have implicated PAI-1 in adipose tissue (AT) expansion while also contributing to insulin resistance. As inflammation is also known to occur in AT during obesity, we hypothesized that in a high-fat diet (HFD)-induced obese mouse model PAI-1 contributes to macrophage-mediated inflammation and metabolic dysfunction.

**Methods:** Four- to five-weeks-old male C57B6/6J mice were fed a HFD (45%) for 14 weeks, while age-matched control mice were fed a standard laboratory chow diet (10% fat). Additional studies were performed in PAI-1 knockout mice and wild type mice treated with an inhibitor (PAI-039) of PAI-1. Macrophage polarization were measured by real time PCR.

**Results:** HFD mice showed increased expression of PAI-1 in visceral white AT (WAT) that also displayed increased macrophage numbers. PAI-1 deficient mice exhibited increased numbers of anti-inflammatory macrophages in WAT and were resistant to HFD-induced obesity. Similarly, pharmacological inhibition of PAI-1 using PAI-039 significantly decreased macrophage infiltration in WAT and improved metabolic status in HFD-induced wild-type mice. Importantly, the numbers of M1 macrophages appeared to be increased by the HFD and decreased by either genetic PAI-1 depletion or PAI-039 treatment.

**Conclusions:** Collectively, our findings provide support for PAI-1 contributing to the development of inflammation in adipose tissue and explain the mechanism of inflammation modulated by PAI-1 in the disordered metabolism in HFD-induced obesity.

## Introduction

Adipose tissue is a highly active metabolic and endocrine organ. In humans and other mammals, adipose tissue can be classified into two subtypes with opposing functions: white adipose tissue (WAT) and brown adipose tissue (BAT). Accumulating evidence indicates that in obese states WAT, in particular, contributes to inflammation, and, compared to lean tissue, shows a higher level of secretion of inflammatory cytokines, such as TNF-α and IL-6 (Hotamisligil et al., 1993; Xu et al., 2003). Substantial interest has developed in the role of various immunocytes including adipose tissue macrophages (ATMs), which exhibit at least two distinct phenotypes (i.e., classically activated M1 “inflammatory” macrophages and alternatively activated M2 “anti-inflammatory” macrophages). Increased numbers of macrophages are found in adipose tissue during obesity (Weisberg et al., 2003) and are considered to be involved in the pathogenesis of components of the metabolic syndrome, including insulin resistance (Hardy et al., 2001; Blüher, 2010). Furthermore, during the progression of high-fat diet (HFD)-induced obesity, the relative numbers of M1 to M2 macrophages in adipose tissue appears to play an important role in the pathogenesis of metabolic dysfunction (Lumeng et al., 2007; Fujisaka et al., 2009).

Plasminogen activator inhibitor-1 (PAI-1) is a key component of fibrinolysis and has been suggested to contribute to increased cardiovascular risk in obese individuals (Kohler and Grant, 2000). PAI-1 is associated with the metabolic syndrome in obesity (Schäfer et al., 2001; Ma et al., 2004; De Taeye et al., 2006) which is characterized by dyslipidemia, hypertension, and glucose intolerance. PAI-1 has also been demonstrated within adipose tissue, with adipocytes being a major source of production for circulating PAI-1 in obese individuals (Folsom et al., 1993; Charles et al., 1998; Eriksson et al., 1998). WAT, specifically visceral adipose tissue, is the major tissue source of PAI-1, accounting for significantly more PAI-1 compared to subcutaneous adipose tissue (Cigolin et al., 1999). Further supporting a relationship between PAI-1 and metabolism, previous studies have shown that PAI-1 deficiency protects against obesity and metabolic dysfunction (Morange et al., 2000). In terms of an interaction with inflammation, macrophage infiltration of WAT leads to increased lipolysis through the increased release of cytokines while TNF-α has been linked to PAI-1 expression in human adipose tissue explants and obese mice (Samad et al., 1999; Alessi and Juhan, 2006). Further supporting a relationship between PAI-1 and tissue macrophage infiltration, increased numbers of macrophages have been shown in lung exudates in the presence of PAI-1, while inhibition of PAI-1 by the administration of a small-molecule PAI-1 inhibitor attenuated infiltration of these cells (Ichimura et al., 2013; Osterholzer et al., 2013; Rebalka et al., 2015). Thus, collectively, these prior observations suggest a possible role for AT PAI-1 in the progression obesity and point to a need to clearly define such relationships.

Given that PAI-1 is dramatically up-regulated in human obesity (Ma et al., 2004), and can be increased by high fat feeding (1–6 weeks) of normal weight subjects, we hypothesized that PAI-1 contributes to macrophage-mediated inflammation in AT and metabolic dysfunction in a HFD-induced obese mouse model. Our results identify a previously unrecognized role for PAI-1 in regulating M1 macrophage numbers in WAT. PAI-1 deficient mice exhibit increased numbers of anti-inflammatory macrophages in WAT and are resistant to HFD-induced obesity. Similarly, pharmacological inhibition of PAI-1 using PAI-039 significantly decreased macrophage infiltration in WAT and improved the metabolic status of HFD-fed wild-type mice. Importantly, genetic loss or inhibition of PAI-1 reduced the apparent numbers of M1-macrophages compared to that observed in HFD-fed wild-type mice. Together, our findings suggest that PAI-1 plays a role in the development of inflammation within adipose tissue and contributes to disordered metabolism during HFD-induced obesity.

## Methods

### Animals

C57BL/6J mice were from purchased from Chongqing Medical University Animal Center, Chongqing, China. PAI-1-deficient (*Pai1*^−/−^) mice were a gift from Dr. Peter Carmeliet, University of Leuven, Leuven, Belgium (Carmeliet et al., 1993). All animal use protocols were reviewed and approved by the Animal Care Committee of Southwest Medical University in accordance with Institutional Animal Care and Use Committee guidelines.

### High fat diet-fed mouse model

Four- to five-weeks-old male C57B6/6J mice were fed a commercially available high-fat diet (HFD; 45% fat by kcal; D12451; Research Diet, New Brunswick, NJ) for 14 weeks, as described previously (Hazarika et al., 2007). Age-matched male mice fed a standard laboratory normal chow diet served as controls. Blood glucose levels were tested from tail vein blood samples using a glucometer (Accu-Check; Roche Diagnostics, Mannheim, Germany). Body weight was measured every 3 days. At the completion of the study, blood was collected under fasting conditions and centrifuged at 1,500 × g for 10 min for measurement of plasma levels of low-density lipoprotein (LDL), high-density lipoprotein (HDL), total cholesterol (TC), and triglycerides (TG) using using an AU680 analyzer (Beckman Coulter, Indianapolis, IN).

### Administration of PAI-039

PAI-039 is an orally administered, specific inhibitor of active PAI-1 (Elokdah et al., 2004). After C57B6/6J mice were fed a high-fat diet for 14 weeks, mice were treated with PAI-039 (2 mg/kg/day; dissolved in vehicle consisting of sterile water containing 0.5% methylcellulose and 2% Tween 80), or vehicle control, treated for 30 consecutive days by twice daily oral gavage (Wu et al., 2015).

### Measurement of plasma PAI-1

Plasma samples were prepared by centrifugation. PAI-1 antigen was measured using a mouse PAI-1 total antigen assay ELISA kit (Molecular Innovations).

### Quantitative real-time PCR

Epididymal white adipose tissue (eWAT) and interscapular brown adipose tissue fat samples were harvested from male ND-WT, HFD-WT, ND-*Pai-1*^−/−^, HFD-*Pai-1*^−/−^, and HFD-WT treated with PI-039, respectively. *N* = 9 mice per group. RNA was extracted using TRIzol reagent (Invitrogen). RNA samples were pre-treated with deoxyribonuclease I (Invitrogen Life Technologies) and cDNA synthesized using a SuperScript kit (Invitrogen Life Technologies). Each sample was analyzed in duplicate with ribosomal 18S RNA used as an internal control. Fold changes in gene expression were determined using the 2^−ΔΔCT^ method. Results are presented as mean ± SEM. Sequences of all primers are shown in Supplementary Table 1.

### Glucose and insulin tolerance testing

After an overnight fast, glucose tolerance tests (GTT) were evaluated following intraperitoneal (IP) injection of D-glucose (Roth, Karlsruhe, Germany; 2 g of glucose/kg lean body mass). After a 4-h fast, insulin tolerance testing (ITT) was performed using IP injections of insulin (0.75 U insulin/kg lean body mass). Blood samples were obtained from the tail, and blood glucose levels measured at 0, 30, 60, and 120 min after glucose injection using an Accu-Check glucometer (Roche Diagnostics).

### Histological assessment

Mouse eWAT was obtained and fixed in 4% (wt/vol) paraformaldehyde in PBS for 3 h and subsequently transferred to 30% (wt/vol) sucrose overnight. The samples were then embedded in OCT compound, frozen, and serially sectioned (6 μm). Cross-sections were prepared for immunofluorescence analysis. Macrophages were determined by immunostaining using anti-F4/80 (Abcam, Cambridge, UK) antibody. Goat anti-rabbit IgG Alexa Fluor 568-conjugated antibody (Molecular Probes, Invitrogen) was used as the secondary antibody. Images were captured using a fluorescence microscope (Leica, Germany). Numbers were quantified in five microscopic fields in each of three cross-sections of each tissue using ImagePro Plus software. In some experiments, cross-sections were stained with hematoxylin-eosin (HE).

### Statistical analysis

Data are presented as the mean ± SEM. Glucose excursions during the GTT were calculated using Microsoft Excel and expressed as incremental area under the curve (iAUC). Differences between groups were analyzed by Student's *t*-test (comparisons of two groups) or analysis of variance (ANOVA; multiple comparisons) using GraphPad Prism (La Jolla, CA, USA). *P* < 0.05 was considered to represent statistical significance.

## Results

### Genetic deletion and pharmacological inhibition of PAI-1 protects from HFD-induced weight gain and associated metabolic disturbances

**Figure 1:**
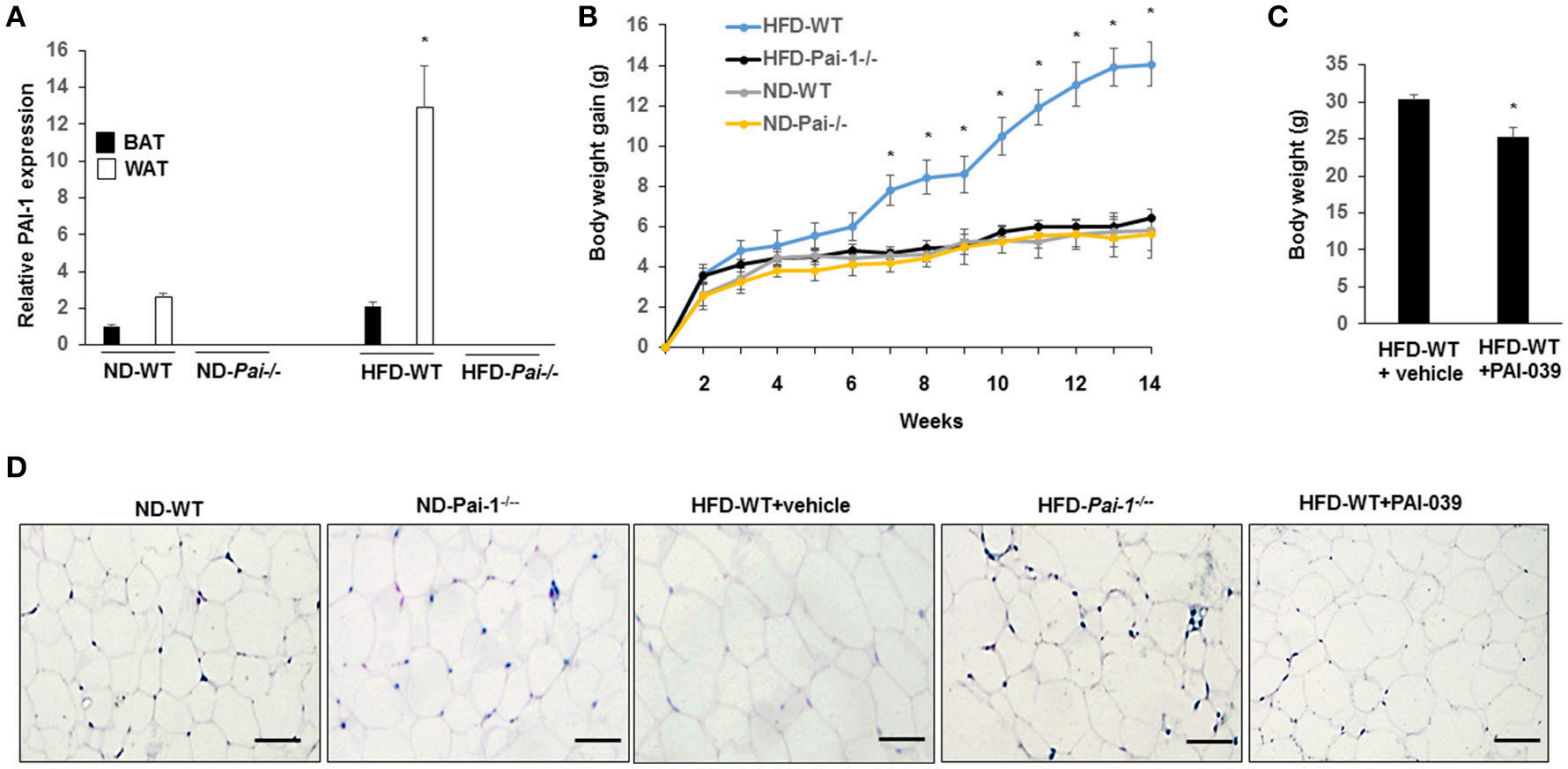
Genetic deletion and pharmacological inhibition of PAI-1 protects from HFD-Induced Weight Gain and associated metabolic disturbances. **(A)** Expression level of PAI-1 in brown adipose tissue (BAT) and white adipose tissue (WT) of normal chow diet-fed WT (ND-WT), ND-*Pai-1*^−/−^, high fat chow-fed WT (HFD-WT), and HFD-*Pai-1*^−/−^ mice. *P < 0.05 vs. ND-WT. **(B)** Changes in body weight of male *Pai-1*^−/−^ (*n* = 9) and their littermate controls (WT, *n* = 13) after being fed an HFD for *Pai-1*^−/−^ 14 weeks. Initial body weights were 23.6 ± 0.16 g for WT and 18.2 ± 0.78 g for *Pai-1*^−/−^ mice. *P < 0.05 vs. HFD-*Pai-1*^−/−^ mice. **(C)** Body weight was assessed by PAI-039 treatment after 30 days in HFD-WT mice. *P < 0.05 vs. vehicle. **(D)** Representative histological images of epididymal white adipose tissue (eWAT) of ND-WT, ND-*Pai-1*^−/−^, HFD-WT, and HFD-*Pai-1*^−/−^ mice.

Significantly higher levels of PAI-1 mRNA were expressed in eWAT compared with those of BAT, both at baseline and after the HF diet (Figure 1A). When fed with normal diet, *Pai-1*^−/−^ and WT mice exhibited a similar weight gain. On feeding of the HF diet, body weight gain was inhibited in *Pai-1*^−/−^ mice (Figure 1B). Circulating PAI-1 concentration was significantly higher in HFD-WT mice (3.02 ± 0.12 ng/mL) than in ND-WT mice (0.95 ± 0.13 ng/mL; *P* < 0.05; *n* = 3 per group).). Circulating PAI-1 concentration was significantly higher in HFD-WT mice (3.02 ± 0.12 ng/mL) than in ND-WT mice (0.95 ± 0.13 ng/mL; *P* < 0.05; *n* = 3 per group). In separate studies the small molecule PAI-1 inhibitor, PAI-039, was used to further characterize the effects of PAI-1 inhibition on body weight, and lipid metabolism in HFD-WT mice. PAI-039 treatment for 30 days caused a significant reduction in circulating PAI-1 concentrations (1.42 ± 0.18 vs. 3.02 ± 0.12 ng/mL, *P* < 0.05; *n* = 3 per group), and a significantly lower body weight compared to HFD-WT mice (25.2 ± 1.28 vs. 31.6 ± 0.66, *p* < 0.05; Figure 1C). Plasma cholesterol levels were similar in ND-WT (1.68 ± 0.12 mM; *n* = 3) and ND- *Pai-1*^−/−^ mice (1.44 ± 0.21 mM; *n* = 3) while levels were elevated in HFD-WT (2.86 ± 0.09 mM; *n* = 3). Cholesterol levels in HFD- *Pai-1*^−/−^ mice (1.31 ± 0.26 mM; *n* = 3) and PAI-039 treated mice (1.72 ± 0.44 mM; *n* = 3) were similar to controls. Plasma lipid characteristics of each experimental group are shown in Table 1.

**Table 1:** Plasma lipid characteristics.

	**ND-WT**	**ND-*Pai-1^−/−^***	**HFD-WT**	**HFD-*Pai-1^−/−^***	**HFD-WT + PAI-039**
TC	1.91 ± 0.12	1.44 ± 0.21^*^	2.86 ± 0.09	1.31 ± 0.30^#^	1.72 ± 0.43^#^
TG	0.27 ± 0.08	0.26 ± 0.05	0.34 ± 0.02	0.26 ± 0.05^#^	0.30 ± 0.02^#^
LDL-C	0.4 ± 0.03	0.45 ± 0.05	0.68 ± 0.08	1.23 ± 0.14^#^	0.39 ± 0.06^#^
HDL-C	1.13 ± 0.11	0.85 ± 0.12^*^	1.63 ± 0.08	0.98 ± 0.08^#^	1.17 ± 0.26^#^

*All units are mM. Values are mean ± SEM*.

* p < 0.05 vs. WT mice receiving normal diet;

# *p < 0.05 vs. WT fed western diet; n = 3 mice per group. TC, total cholesterol; TG, triglycerides; LDL-C, low-density lipoprotein cholesterol; HDL-C, high-density lipoprotein cholesterol; WT, wild-type*.

WAT plays a key role in the regulation of adiposity and energy metabolism and has been found to undergo significant metabolic changes during feeding of a high-fat diet (Hazarika et al., 2007). Specifically, adipocyte hypertrophy and hyperplasia occurs in HFD-induced obesity. Consistent with results reported previously for chow-fed animals (Kohler and Grant, 2000), adipocytes in ND- and HFD- *Pai-1*^−/−^ mice were smaller in size compared with adipocytes from HFD-WT mice after 14 weeks of HFD feeding. Similarly, HFD-WT mice treated with PAI-039 had decreased adipocyte size compared with HFD-WT mice (Figure 1D). Group data showing quantification of adipocyte areas are shown in Supplementary Figure 1.

### Genetic deletion and pharmacological inhibition of PAI-1 improves glucose tolerance and insulin sensitivity

**Figure 2:**
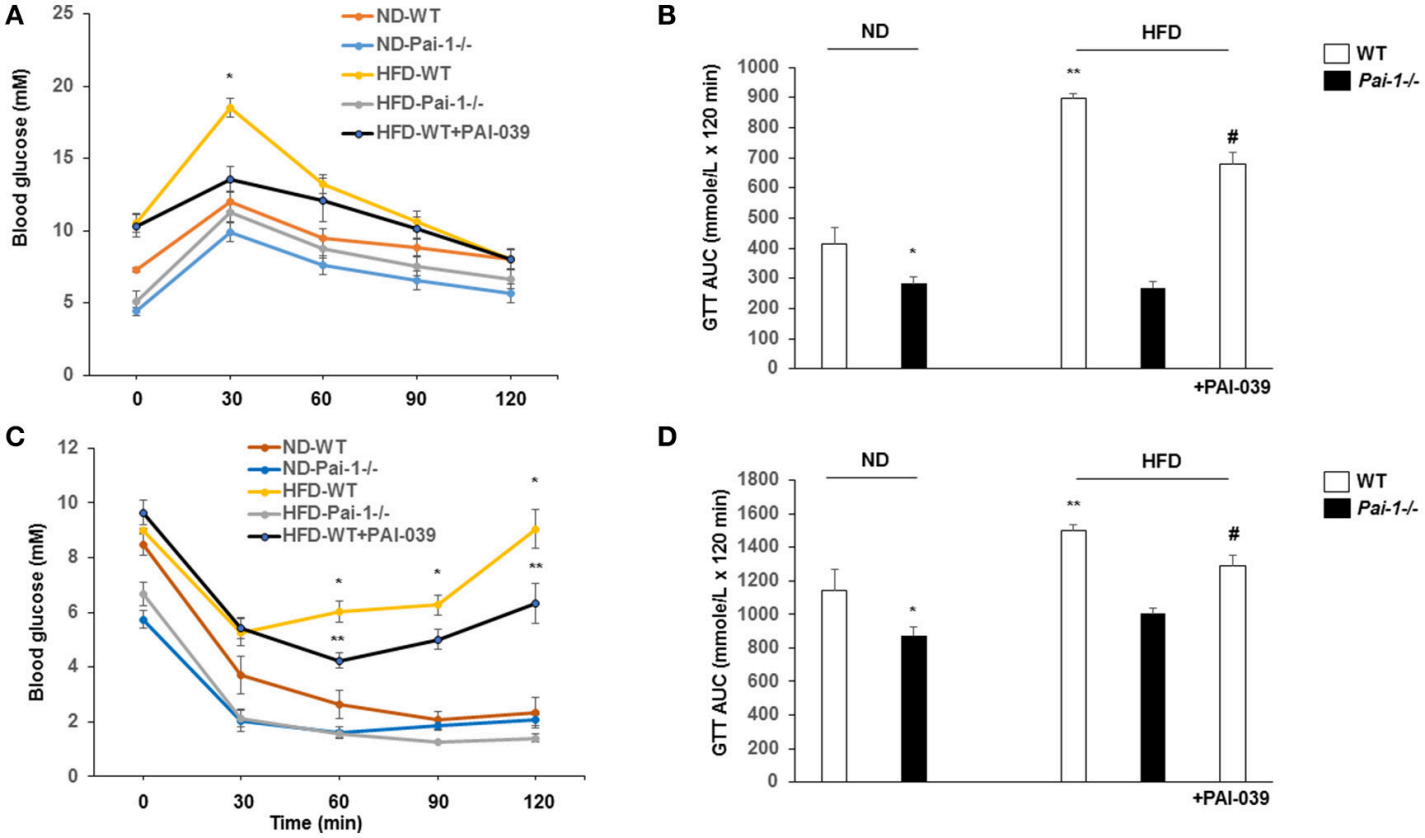
Genetic deletion and pharmacological inhibition of PAI-1 improves glucose tolerance and insulin sensitivity. **(A,B)** Glucose tolerance tests (GTT) and AUC in each group. *N* = 6 per group. *P < 0.05 vs. ND-WT; **P < 0.05 vs. ND-WT; ^#^*P* < 0.05 vs. HFD-WT vehicle. HFD was associated with elevated fasting glucose levels and impaired glucose tolerance. PA1-1 depletion or inhibition **(C,D)** Insulin tolerance tests (ITT) and AUC in each group. *N* = 6 per group. *P < 0.05 vs. ND-WT; **P < 0.05 vs. ND-WT; ^#^*P* < 0.05 vs. HFD-WT.

After 14 weeks on the HFD, WT mice developed hyperglycemia while deletion of PAI-1 was associated with fasting plasma glucose levels within the normal range (8.0 ± 0.3 vs. 12.3 ± 0.5 mM, *p* < 0.05). Treatment of HFD-WT mice with PAI-039 for 30 days resulted in decreased plasma glucose levels compared to HFD-WT mice (5.7 ± 0.3 vs. 12.2 ± 1.2 mM; *p* < 0.05). Intraperitoneal glucose and insulin tolerance tests were performed to further characterize the metabolic state of the animal groups. Prior to each test, fasting plasma glucose levels were significantly (*p* < 0.05) lower in ND and WDF PAI-1^−/−^ mice compared to ND-WT (Figure 2) mice perhaps consistent with previously reported increases in insulin sensitivity and metabolic rate in the knockout model (Ma et al., 2004). HFD feeding caused impaired glucose tolerance (Figures 2A,B) and decreased insulin sensitivity (Figures 2C,D). Both HFD- *Pai-1*^−/−^ and PAI-039 treated mice showed improved glucose tolerance (Figures 2A,B) and attenuated apparent insulin resistance (Figures 2C,D) despite being similarly fed the HFD.

### Macrophage quantification in white adipose tissue

**Figure 3:**
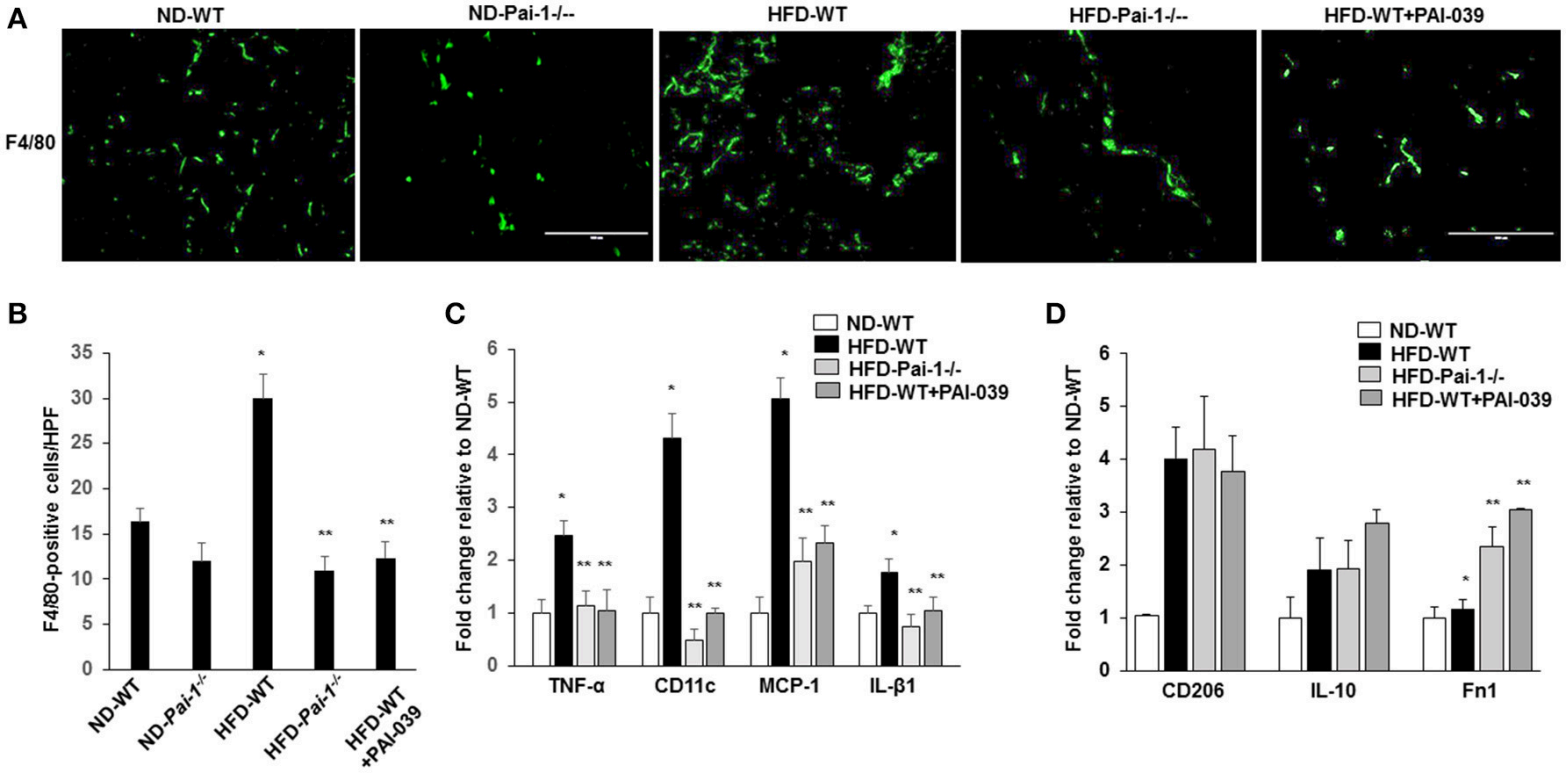
Macrophage quantification in white adipose tissue. **(A)** Representative images of macrophages as assessed by F4/80 Immunofluorescence in eWAT from ND-WT, ND-*Pai-1*^−/−^, HFD-WT, HFD-*Pai-1*^−/−^, and PAI-039-treated mice. Scale bars, 100 μm. HPF: high power field. **(B)** Quantification of anti-F4/80 positive-macrophage infiltration of eWAT. *P < 0.05 vs. ND-WT; **P < 0.05 vs. HFD-WT. **(C,D)** The gene profile of the M1 **(C)** and M2 **(D)** phenotype as assessed by quantitative RT-PCR of eWAT in ND-WT, HFD-WT, HFD-*Pai-1*^−/−^, and PAI-039-treated mice, respectively. All bars show Mean ± SEM. Data are mean of three experiments and are expressed as fold-control. *P < 0.05 vs. ND-WT; **P < 0.05 vs. HFD-WT.

Previous studies have reported that feeding of a HFD results in the accumulation of macrophages in WAT, which can subsequently contribute to inflammation (Elokdah et al., 2004). Consistent with this we found a significant increase in the total number of macrophages (F4/80-positive) in WAT from HFD-WT mice compared to ND-WT mice (Figures 3A,B). PAI-1 deficiency and PAI-039 treatment was associated with a significant reduction in macrophage numbers in HFD-fed mice compared to their respective control groups (Figures 3A,B). As an indicator of the effect of PAI-1 on the polarization of resident macrophages in WAT, transcript markers for the M1 inflammatory macrophage phenotype were assessed using quantitative RT-PCR analysis. The pro-inflammatory M1 markers, including TNF-α, MCP-1, IL-β1, and CD11c, exhibited a significant decrease in HFD- *Pai-1*^−/−^ mice and PAI-039 treated HFD-WT mice compared to HFD-WT mice (Figure 3C). Further, expression levels in HFD- *Pai-1*^−/−^ mice and PAI-039-treated HFD-WT mice were similar to those found in ND fed mice. Meanwhile, we examined the anti-inflammatory M2 markers, including CD206, IL-10 and Fn1, and levels of CD206 and IL-10 in HFD- *Pai-1*^−/−^ mice and PAI-039-treated HFD-WT mice were similar to those found in HFD fed mice. The Fn1 was significantly increased in HFD- *Pai-1*^−/−^ mice and PAI-039 treated HFD-WT mice compared to HFD-WT mice (Figure 3D). These data were thus taken to indicate that either PAI-1 deficiency or pharmacological inhibition of PAI-1 can prevent a HFD-induced infiltration of macrophages into WAT and, more specifically, decrease numbers of the pro-inflammatory M1 phenotype.

## Discussion

Growing evidence indicates that inflammation plays an important role in the pathological processes contributing to obesity and its complications (Chawla et al., 2011). Further, it has been suggested that an abnormal increase in numbers of pro-inflammatory macrophages gives rise to local inflammation in adipose tissue during obesity (Weisberg et al., 2003; Xu et al., 2003). Relevant to the present studies PAI-1 has been reported to have important physiological and pathophysiological roles some of which affect macrophage migration (Cao et al., 2006). PAI-1 has also been shown to be expressed in WAT and linked to metabolic dysfunction (Alessi and Juhan, 2006; Ichimura et al., 2013). Despite these observations relatively little is known regarding the direct actions of PAI-1 in modulating pro-inflammatory macrophage infiltration into WAT and disease progression during diet-induced obesity/T2D. In the present studies we, therefore, focused on the role of PAI-1 in tissue-specific inflammation and macrophage infiltration in HFD-induced obese mice.

Using both PAI-1 knockout mice and WT mice treated with a pharmacological inhibitor of PAI-1 we initially confirmed prior studies implicating a causative role for PAI-1 in obesity and insulin resistance (Ma et al., 2004) and validated our model in relation to whether PAI-1 contributed to the metabolic abnormalities associated with HFD feeding. Feeding of WT/control mice with a HFD caused a significant gain in body weight. Weight gain, however, was not observed in either *Pai-1*^−/−^ mice or animals treated with the PAI-1 inhibitor. Both HFD- *Pai-1*^−/−^ mice and PAI-039 treated mice showed lower plasma levels of total cholesterol compared with HFD-WT mice, indicative of improved lipid metabolism. Similarly improved glucose tolerance and apparent insulin sensitivity was evident in both HFD- *Pai-1*^−/−^ mice and PAI-039 treated mice. Thus, consistent with earlier observations (Ma et al., 2004), suppression of PAI-1 in mice appears to protect against the development of HFD-induced obesity and associated metabolic dysregulation.

WAT is now recognized to be an active metabolic and endocrine tissue producing adipokines, as well as pro- or anti-inflammatory cytokines capable of regulating lipid metabolism and local inflammation (Amengual et al., 2013). Macrophages infiltrating WAT secrete pro inflammatory factors that promote metabolic dysfunction (Surmi et al., 2010). In obese and diabetic subjects adipose tissue has been shown to contain increased numbers of M1 macrophages, a major source of pro-inflammatory cytokines (Grant and Dixit, 2015). Our results indicate that PAI-1 deficiency or PAI-039 treatment reduces macrophage invasion into WAT. To our knowledge this represents the first report linking PAI-1 to macrophage infiltration into adipose tissue. Of further importance, macrophage infiltration, in a high fat feeding model, could be prevented by both genetic deletion of PAI-1 and administration of a small molecular weight inhibitor of PAI-1 suggesting therapeutic strategies for decreasing the effects of PAI-1 in obese/T2D states.

We further found that PAI-1 deficiency or PAI-039 treatment caused a significant reduction in a subset of M1 macrophage-specific genes in WAT of HFD-fed mice compared to HFD-WT mice. While this is apparent contrast to the studies of Rebalka et al. (Rebalka et al., 2015), who reported that PAI-039 had little effect on M1 macrophages while increasing an M2a subset, their studies used a streptozotocin-induced model of diabetes, examined wound tissue as opposed to adipocytes and used a shorter duration of treatment with the inhibitor. The data from the present study suggest that genetic deletion and pharmacological inhibition of PAI-1 will cause a shift in WAT from an M1-mediated inflammatory environment to a relatively anti-inflammatory condition. Of particular interest, we observed a significant reduction in the level of TNF-α mRNA in the WAT of PAI-1 deficiency or PAI-039 treated HFD-fed mice. TNF-α has previously been shown to be involved in adipose tissue inflammation and insulin resistance during obesity (Boutens and Stienstra, 2016). Collectively, these results suggest that in WAT PAI-1 may regulate glucose hemostasis through modulation of macrophage polarization in WAT, however, additional studies are required to determine specific relationships between alterations in macrophage subpopulations and PAI-1-regulated inflammatory responses regulated during metabolic disorders.

Our study significantly broadens the current understanding of the pathological mechanisms by PAI-1 in adipose tissue during metabolic disorder. There are some limitations to this study. While our study demonstrated that PAI-1 plays an important role in the regulation of adipose tissue dysfunction and glucose homeostasis, limited by the availability of the adipose specific PAI-1 knockout strain. Additional studies are warranted to resolve this important issue. An additional consideration is that the present studies were conducted in male mice so as to initially understand the relationships between PAI-1 and adipose tissue macrophage numbers in the absence of fluctuating levels of sex hormones. Interestingly female mice fed a HFD show an estrogen-induced (mediated through estrogen receptor alpha) increase in PAI-1 expression despite the fact that the estrogen attenuates insulin resistance and glucose intolerance (Riant et al., 2009). In the light of the present results this suggests that there may be apparent sexual dimorphism. As such an important future direction will be to determine the effect of sex on relationships between PAI-1 and infiltration of macrophages into adipose tissue.

In summary, the pathophysiologic mechanisms by which adipose tissue-derived PAI-1 influences the development of metabolic syndrome remain to be determined. Our results demonstrated that the absence of PAI-1 could recruit more macrophages into adipose tissues and link to the promotion of metabolic dysfunction. Further, genetic deletion and pharmacological inhibition of PAI-1 showed that PAI-1 was involved in macrophage polarization in WAT in HFD-induced obesity in mice. However, more evidence should be required to explain the mechanism of inflammation modulated by PAI-1 in WAT during metabolic disorder.

## Author contributions

All authors made substantial contributions to the conception and design of the various aspects of the prospective studies or to the acquisition, analysis or interpretation of data. All authors also contributed to drafting the article or revising it critically for important intellectual content and have given final approval of the version to be published. JW and RL are responsible for the integrity of this work as a whole, including the study design, access to data, and the decision to submit and publish the manuscript.

## Conflict of interest statement

The authors declare that the research was conducted in the absence of any commercial or financial relationships that could be construed as a potential conflict of interest.
