# Supplementary material for: PAI-1 Exacerbates White Adipose Tissue Dysfunction and Metabolic Dysregulation in High Fat Diet-Induced Obesity
Source: Front Pharmacol. 2018 Sep 26;9:1087. doi: 10.3389/fphar.2018.01087 (PMC6169321; doi:10.3389/fphar.2018.01087)
Supplement: Supplementary file 1 [file Table_1.DOCX]

**Supplemental Table 1 Sequences of primers used in the study**

| CD11c | Forward | CACTCAGTGACTGCCCAAAA |
| --- | --- | --- |
| [NM_021334.2](https://www.ncbi.nlm.nih.gov/entrez/viewer.fcgi?db=nucleotide&id=118130485) | Reverse | CCTCAAGACAGGACATCGCT |
| IL-1β | Forward | ACTACAGGCTCCGAGATGAACAAC |
| [NM_008361.4](https://www.ncbi.nlm.nih.gov/entrez/viewer.fcgi?db=nucleotide&id=921274059) | Reverse | CCCAAGGCCACAGGTATTTT |
| IL-6 | Forward | CACATGTTCTCTGGGAAATCG |
| [NM_001314054.1](https://www.ncbi.nlm.nih.gov/entrez/viewer.fcgi?db=nucleotide&id=930945755) | Reverse | TTGTATCTCTGGAAGTTTCAGATTGTT |
| TNF-α | Forward | ACGGCATGGATCTCAAAGAC |
| [NM_001278601.1](https://www.ncbi.nlm.nih.gov/entrez/viewer.fcgi?db=nucleotide&id=518831588) | Reverse | AGATAGCAAATCGGCTGACG |
| MCP-1 | Forward | CTCTTTCCATTTTTGCATCAAGTTC |
| [NM_008354.3](https://www.ncbi.nlm.nih.gov/entrez/viewer.fcgi?db=nucleotide&id=145966810) | Reverse | CCCATCTTTAACCGATCTAGAGTCA |
| IL-10 | Forward | TGTCAAATTCATTCATGGCCT |
| [NM_010548.2](https://www.ncbi.nlm.nih.gov/entrez/viewer.fcgi?db=nucleotide&id=291575143) | Reverse | ATCGATTTCTCCCCTGTGAA |
| TGF-β1 | Forward | TGCTAATGGTGGACCGCAA |
| [NM_011577.2](https://www.ncbi.nlm.nih.gov/entrez/viewer.fcgi?db=nucleotide&id=930697458) | Reverse | CACTGCTTCCCGAATGTCTGA |
| CD206 | Forward | CATGGATGTTGATGGCTACTGGAG |
| [NM_008625.2](https://www.ncbi.nlm.nih.gov/entrez/viewer.fcgi?db=nucleotide&id=224967061) | Reverse | GTCTGTTCTGACTCTGGACACTTG |
| Fn1 | Forward | GGAGTGGCACTGTCAACCTC |
| [NM_001276413.1](https://www.ncbi.nlm.nih.gov/entrez/viewer.fcgi?db=nucleotide&id=449083346) | Reverse | ACTGGATGGGGTGGGAAT |
| GAPDH | Forward | TTCACCACCATGGAGAAGG |
| [NM_008084.3](https://www.ncbi.nlm.nih.gov/entrez/viewer.fcgi?db=nucleotide&id=576080553) | Reverse | CTCGTGGTTCACACCCATC |
