# Supplementary figures and images for: PAI-1 Exacerbates White Adipose Tissue Dysfunction and Metabolic Dysregulation in High Fat Diet-Induced Obesity
Source: Front Pharmacol. 2018 Sep 26;9:1087. doi: 10.3389/fphar.2018.01087 (PMC6169321; doi:10.3389/fphar.2018.01087)

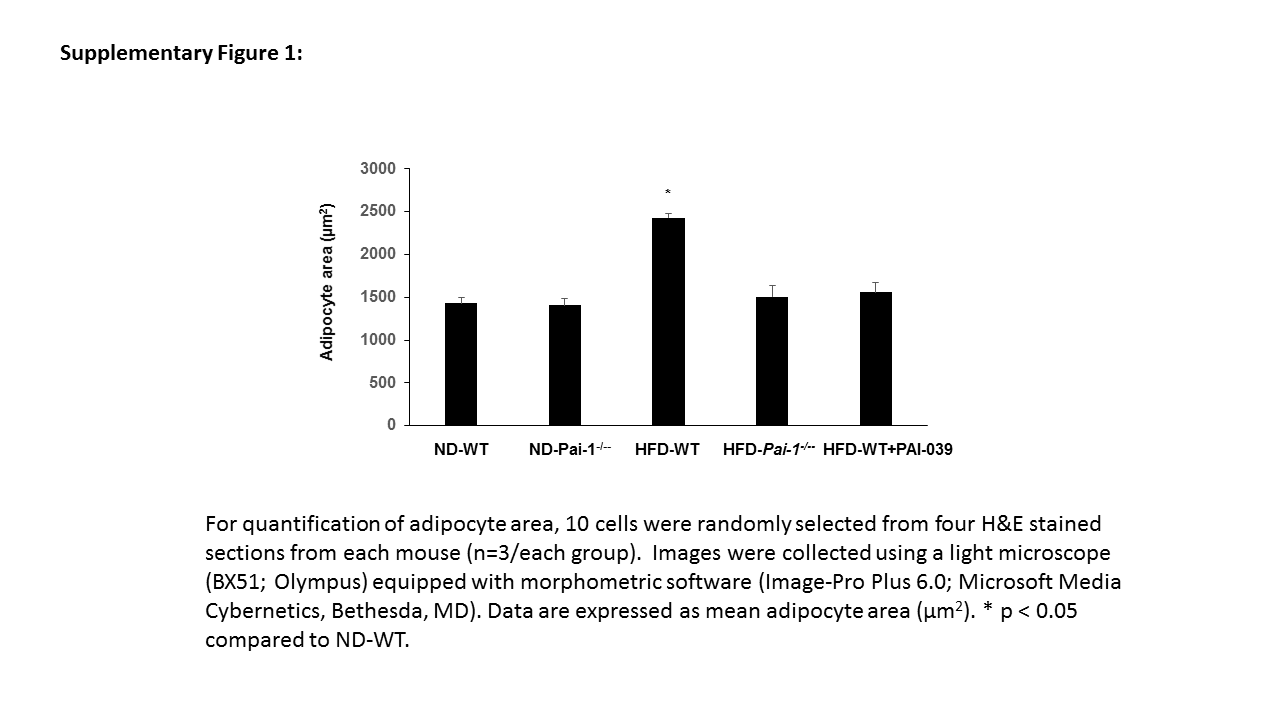

Supplement: Supplementary file 2 [file Image_1.TIF]
